# “Bone-οn-Bone” surgical reconstruction of moderate severity, flexible single curve adolescent idiopathic scoliosis: continuing improvements of the technique and results in three scoliosis centers after almost twenty years of use

**DOI:** 10.1186/s13013-015-0032-0

**Published:** 2015-03-24

**Authors:** Robert W Gaines, Kan Min, Daniel Zarzycki

**Affiliations:** Columbia Orthopaedic Group, 1 South Keene Street, MO 65201 Columbia, USA; Department of Orthopaedics, Balgrist Clinic, University of Zurich, Forchstrasse 340, 8008 Zurich, Switzerland; Orthopaedic and Rehabilitation University Hospital Collegium Medicum Jagiellonian University, Zakopane, Poland

## Abstract

The “bone-on-bone” reconstruction for adolescent idiopathic scoliosis is reviewed in this article. Extensive use over the past 18 years has identified it’s functional benefits outstanding clinical results, and very limited complications. This is an extensive update of it’s application, since it’s introduction, 18 years ago.

**Electronic supplementary material:**

The online version of this article (doi:10.1186/s13013-015-0032-0) contains supplementary material, which is available to authorized users.

## ᅟ

Surgeons, and other readers who are familiar with adolescent idiopathic scoliosis can get a prompt, simple education about the "bone-on-bone" procedure by watching the short video (“Additional file. Double-click here for it to play”) which accompanies this article.

## Introduction

Optimal surgical correction of single curve adolescent idiopathic scoliosis reduces the primary curve to less than 30 degrees, and reduces and balances the compensatory curves. It normalizes the sagittal plane alignment within the operated area and the spine as a whole, while operating as few vertebral segments as possible, to preserve mobility and optimal spinal flexibility. The functional limitations imposed by long spinal fusions have been well documented by Lonner, Sucato and d’Andrea. The chosen operative procedures must also preserve pulmonary function.

These goals are nicely provided by the “bone-on-bone” surgical procedure which was first performed in 1996 and first published by Brodner and Gaines, et. al. in 2003 [1] and again in by Kusakabe and Gaines, et. al. in 2009 [2].

The first publication by Dr. Zarzycki was in 2007 [3]. He and his large group of mostly young surgeons from Zakopane, Poland have performed over 1300 “bone-on-bone” cases since 2003 with very fine clinical results, few implant problems, non-unions or re-operations.

Dr. Min developed a mini-thoracotomy approach which has eliminated the concern regarding damage to pulmonary function by anterior-based surgical approaches for the correction of adolescent idiopathic scoliosis. He published this approach in 2012, after developing it in the mid-90′s [4].

### Evolution of the procedure

Dwyer [5] introduced the use of the anterior approach, multiple discectomy, anterior fusion and anterior instrumentation for the correction of adolescent idiopathic scoliosis. His innovation was the introduction of a new titanium staple and cable implant system. He emphasized “thorough” discectomy, and care removing the posterior longitudinal ligament to avoid dural and spinal cord injury, but did not certainly perform or recommend “total discectomy” as we currently recommend and perform during the “bone-on-bone” procedure. He also routinely added bone chips from the exposure rib into the disc space following curve correction.

Hall and many others adopted the Dwyer system after it’s introduction in 1969. In 1981, twelve years following the introduction and use of the Dwyer implant system, Hall [6] reported better results, with fewer non-unions and implant problems, after “more thorough removal of disc-space material, particularly along the posterior margin of the disc space.” He also mentions that over- correction helped, on occasion, to balance the entire spine. In a book chapter in 1997 [7], he mentions removal of the disc back to, and including the posterior annulus, but leaving the posterior longitudinal ligament intact. He again reinforces the importance of gaining overcorrection of some operated curves.

Zielke [8] introduced a revised staple, and a threaded 3 mm rod, instead of a cable, and a new mechanical derotation device. He, also, suggested removal of the discs “back to the posterior longitudinal ligament”, and also placed bone chips into the residual disc void following correction of the curve. He suggested avoiding dural injury, as well.

Kaneda [9] introduced a dual rod, dual staple and screw system after numerous rod and cable fractures had been identified in reported series of cases using the Dwyer cable and the Zielke threaded rod systems. Kaneda also emphasized “thorough” discectomy and removal of the concave annulus at the resected levels. However, he did not recommend the “total discectomy” that we have used and recommend for the “bone-on- bone” procedure. Like all the authors before him, Kaneda recommended inserting bone chips from the exposure rib before completing correction of the curvature by inserting the screws and achieving correction of the curve.

Dickson [10] performed posterior instrumentation with square-ended Harrington rods for single curves less than 60 degrees, but emphasized correcting the thoracic lordosis of the apical segments with concave side sublaminar wires. For cases between 60 and 90 degrees, anterior “thorough” discectomies were performed during a first stage procedure, but the instrumentation was performed posteriorly with sublaminar wires at a second stage. In addition, the levels for the posterior instrumentation were from “end vertebra to end vertebra” of the Cobb angle on the pre-operative standing film, not shorter than the measured levels on the standing film. These procedures were called the Leeds Procedures.

### Uniquenesses of the “Bone-On-Bone” procedure

The first key modification of the procedure which differentiates “bone-on-bone” procedure from it’s predecessors is the “total discectomy” of the apical segments.

Previous discectomies performed to correct adolescent idiopathic scoliosis were focused on removing the anterior and central portions of the apical discs. The posterior annulus was not entirely removed. Removal of the posterior annulus, and then achieving “bone-on-bone” apposition over the fusion segment is the distinctive structural difference between the “bone-on-bone” procedure and the procedures which predated it’s introduction.

The second key modification is simultaneous anterior short-segment instrumentation over only the apical segments—which are identified by performing a “stretch film” and identifying and subsequently removing the discs and then instrumenting the vertebral bodies contained within the “Cobb angle on the stretch film”.

The third key modification we introduce is the use of the mini-thoracotomy approach developed by Dr. Min [4]. This modification prevents any limitation of the post-operative pulmonary function of the patient. It also permits full closure of the pleura, following the instrumentation.

### Analyzing a scoliosis patient in preparation for a “bone-on-bone” procedure

Pre-operative stretch or bending films allow the operating surgeon to identify the apical 3–5 motion segments which are the most deformed. These discs are totally removed during the surgical reconstruction. This permits the straightening of these maximally deformed segments and allows the compensatory curves to return to more normal alignment. The “total discectomy” also usually allows restoration of the sagittal plane alignment of the involved levels to return to normal, once “bone-on- bone” apposition is achieved.

Spinal implants are applied following the “total discectomies” at the apical levels to bring the vertebrae on either side of the discs into intimate “bone-on-bone” apposition, which permits prompt healing following the reconstruction.

### Selecting ideal cases

**Figure 1:**
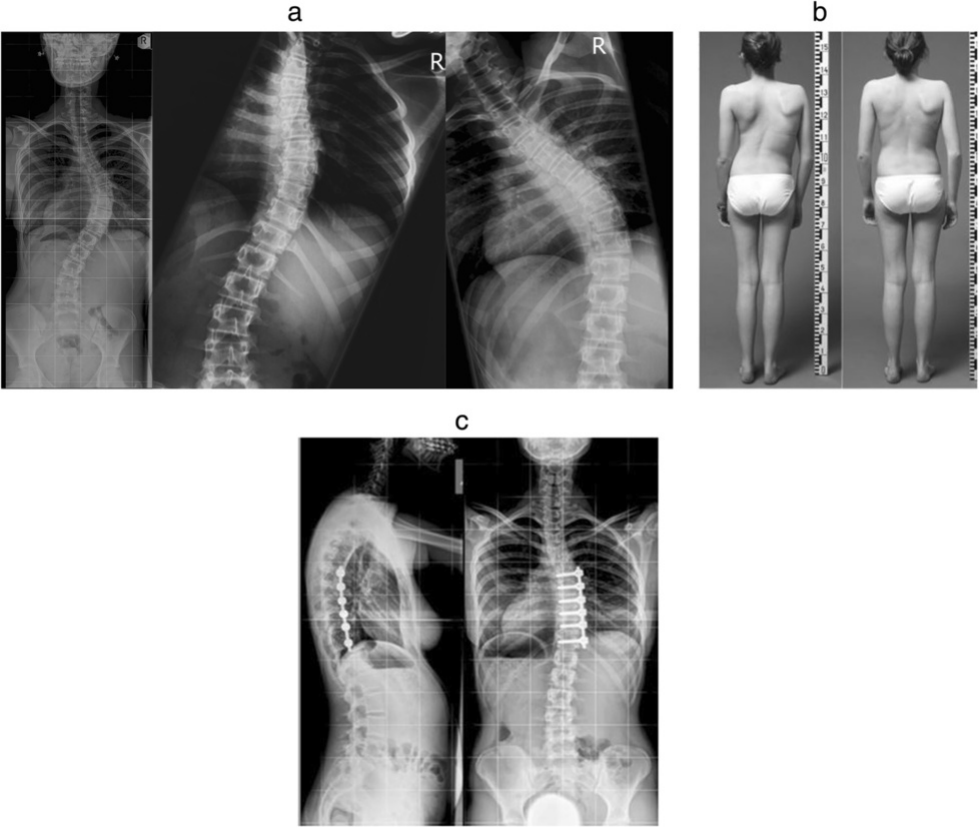
**a: A single thoracic curve (Lenke 1).** Disc T11/T12 opens in supine bending. T11 is distal LIV. In anteroposterior standing radiograph the apex is disc T8/T9. There are three vertebrae (T9, T10,T11) distal to the apex and therefore three proximal vertebrae (T6, T7, and T8) need to be included in the proximal arm. **b**: Radiographs and clinical pictures preoperatively and at 5-year follow-up.

**Figure 2:**
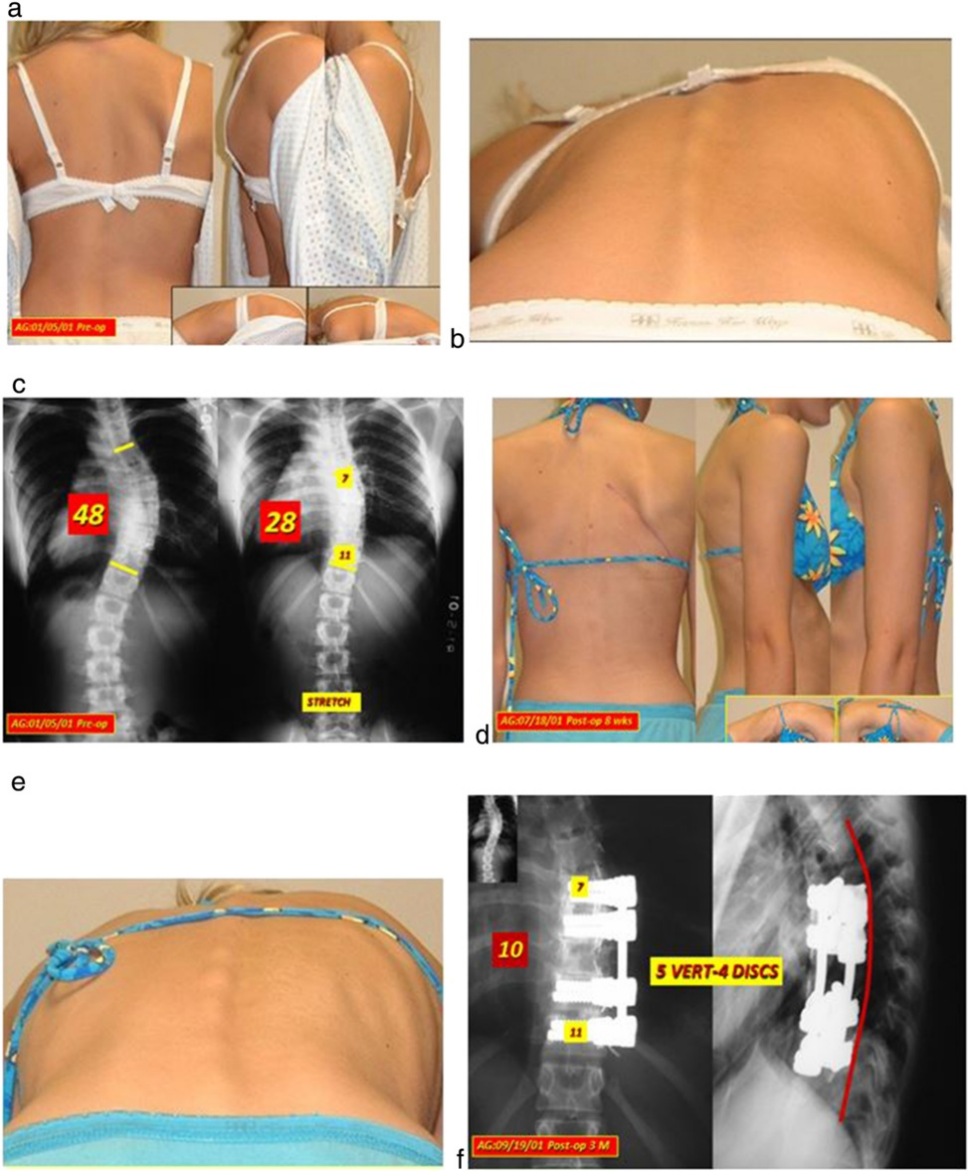
**a: Lenke 1a case,15 year old girl. b**: Significant rib prominence. **c**: 48 degree primary curve reduces to 28 degrees on stretch film. Apical levels (Cobb angle on the stretch film) T7 to T11. **d**: 2 mos. Post-op clinical photos. **e**: rib hump correction. **f**: 10 degree post-op curve with normalized sagittal plane 3 mos. post-op.

**Figure 3:**
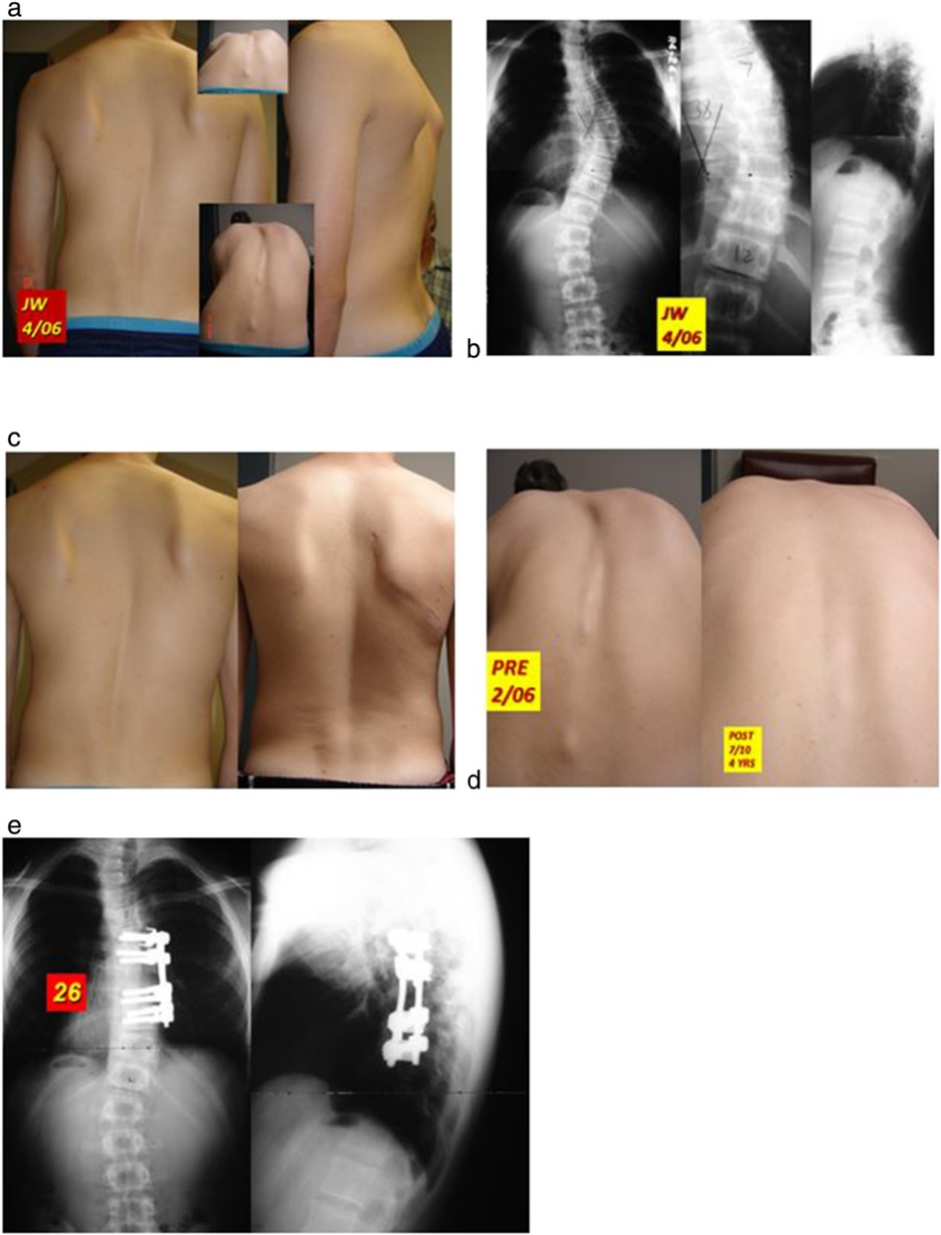
**a: 16 year old boy with Lenke 2a curve. b**: 51 degree primary curve stretches to 38 degrees from T7 to T12. **c**: correction of scoliotic deformity 3 mos. post “bone-on-bone” correction. **d**: rotational correction 4 years post-op. **e**. balance restored in AP and lateral planes by short-segment “bone-on-bone” approach.

**Figure 4:**
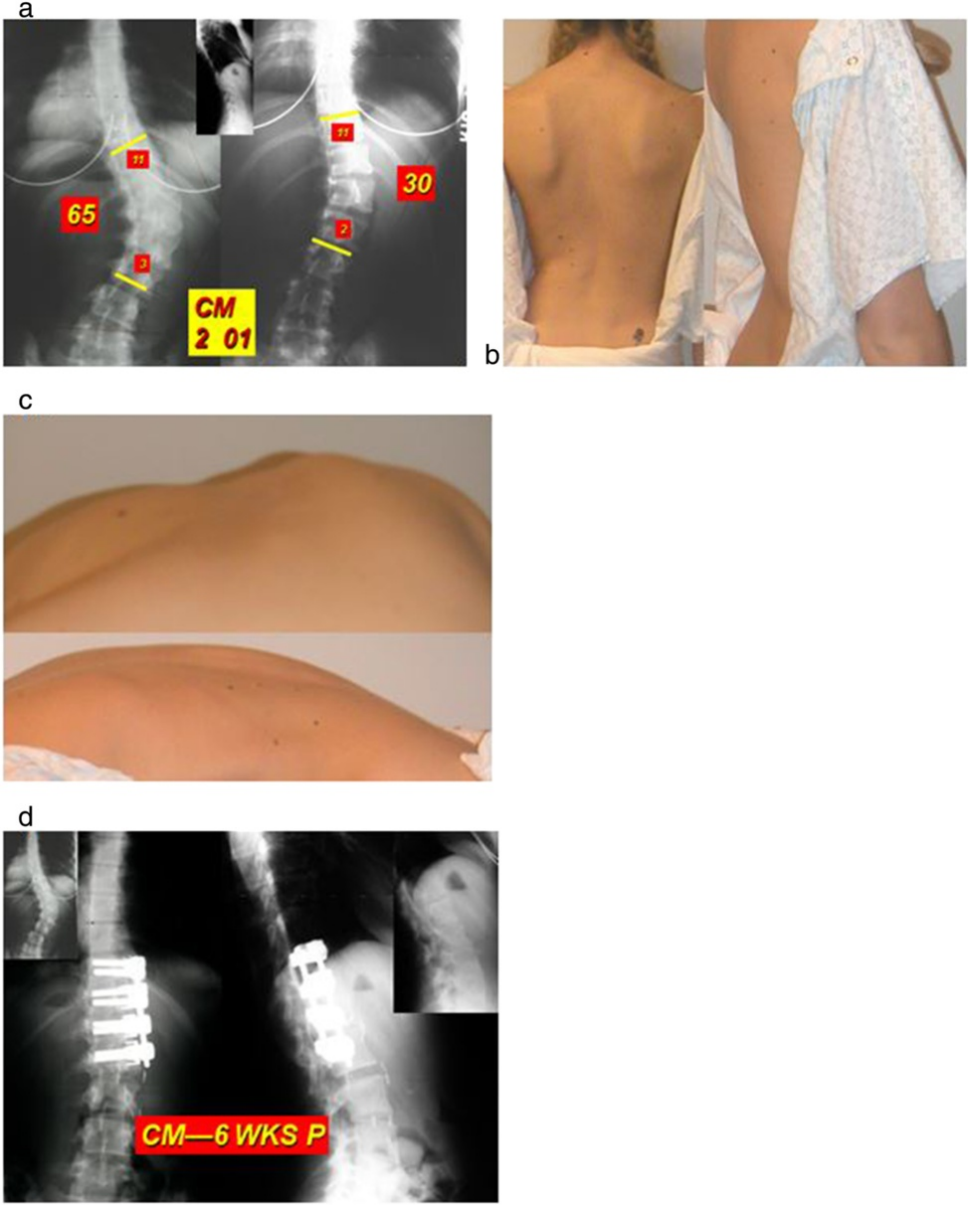
**a: Lenke 5 curve-24 year old woman.** 65 standing goes to 30 on stretch film. Cobb angle on “stretch film” is 30 degrees. Instrumentation will be from T11 to L2. **b**: decompensation to R is obvious. Lumbar hump obvious. **c**: decompensation to R is obvious. Lumbar hump obvious. **d**: correction full and perfect “flat” sagittal plane alignment within the instrumented segment. Interbody cages rarely necessary above L2-3 disc space. However, if they are needed, use them ! Sagittal plane alignment after the surgery is as important as coronal plane correction. BOTH must be perfect post-op.

**Figure 5:**
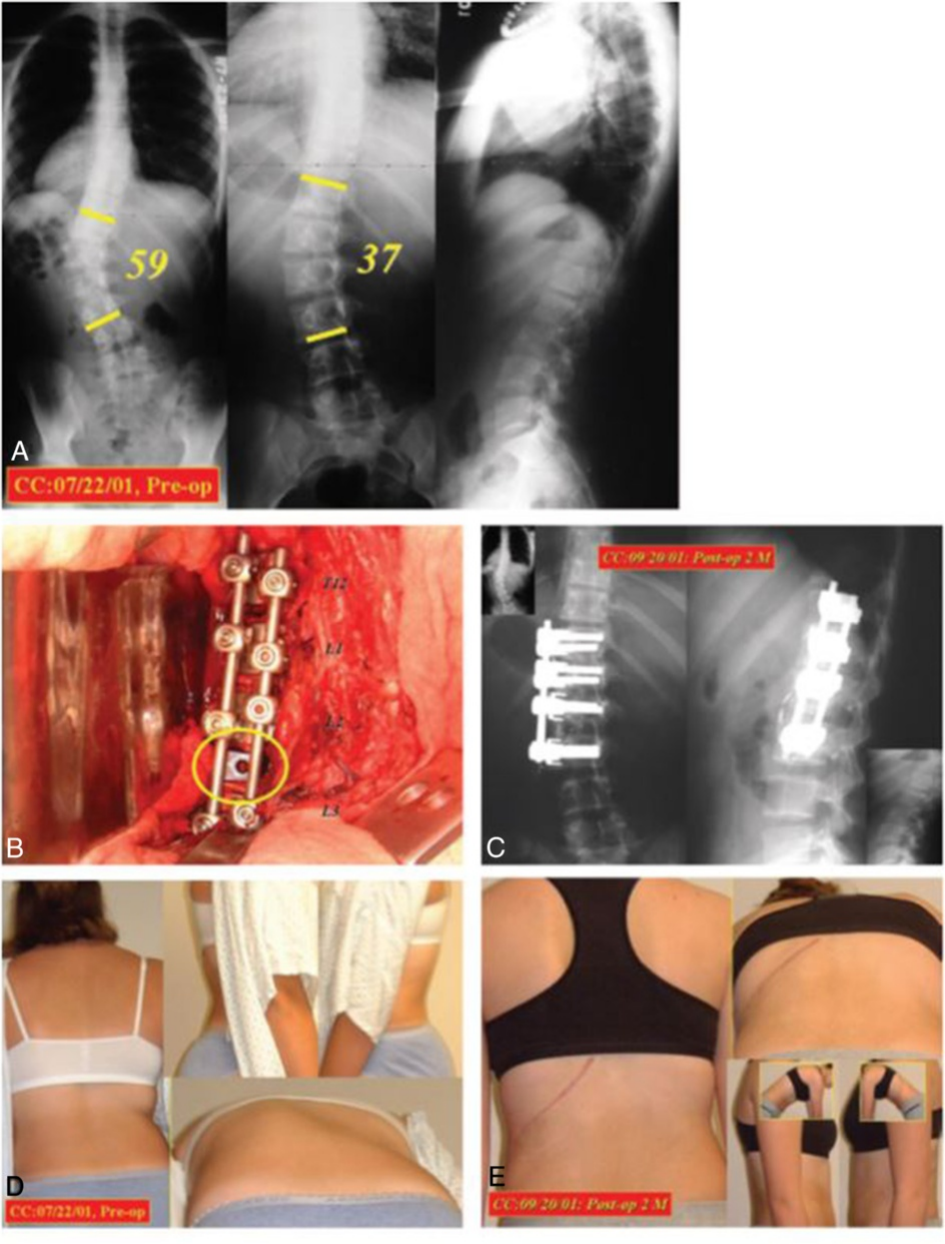
**A, Posteroanterior and lateral preoperative standing radiographs of a 13-year- old girl with a 59° Lenke 5CN left thoracolumbar curve.** The curve is reduced to 37° on the “stretch film.” There is thoracolumbar junctional kyphosis of 7° in the later instrumented segments T12 to L3. **B,** A carbon fiber cage is inserted into the L2–L3 disc space to improve the sagittal alignment. **C,** 2 months after anterior instrumentation, the entire thoracolumbar curve is reduced to 13° with the instrumented levels T12 to L3 improved to 4°. **D,** Presurgical trunk decompensation to the left with trunk rotation. **E,** View 2 months after surgical correction: the trunk is balanced over the pelvis; there is no more trunk rotation.

While the “bone-on-bone” surgical technique can and has been applied to different types of scoliosis, it was developed specifically for the correction of single curve adolescent idiopathic scoliosis (Figures 1, 2, 3, 4, and 5)

.

### Preoperative planning

**Figure 6:**
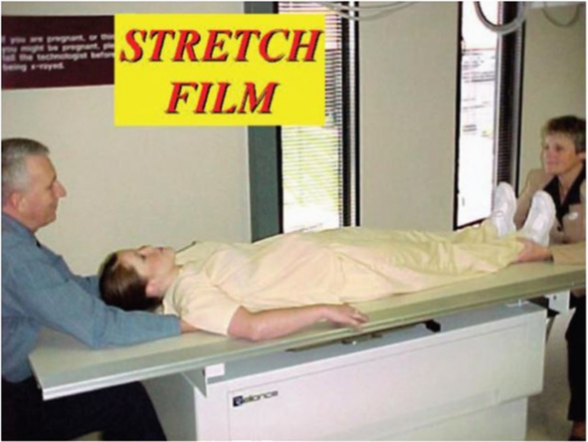
**Performing a “Stretch film”.** The patient is laid supine on the radiograph table and a 36-inch film is placed under the patient. Two trained clinical persons then “stretch” the patient gently while the patient is instructed to relax and enjoy the experience.

**Figure 7:**
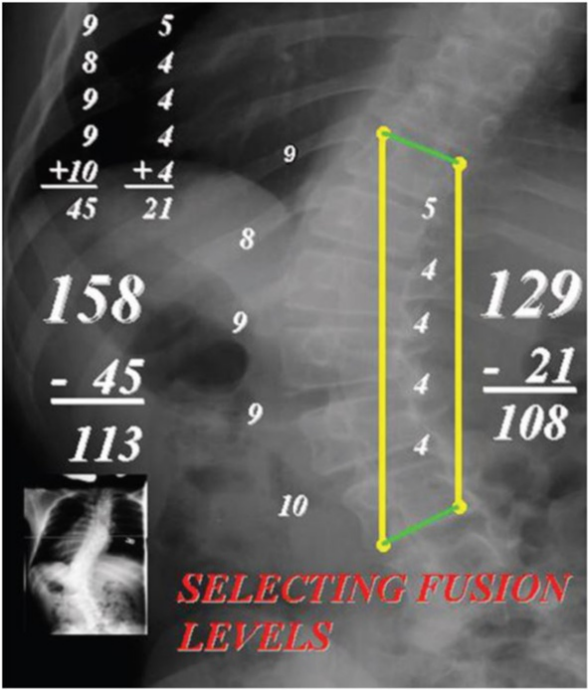
***Why use the “stretch film”*** **.** The fundamental measurement made on the “stretch film” is the Cobb angle of the major curve while the spine is being stretched. The unique value of the “stretch film” is that is clearly identifies the area of major deformity, while the spinal column is compensated, over the pelvis, and the compensatory curves are well corrected. In the author’s experience, this type film is much easier to interpret than bending films, which, by their nature, are NOT made with the patient’s spine fully compensated and the compensatory curves minimized. *Analyzing/measuring the “stretch film”.* From the Cobb angle on the “stretch film,” a measurement (in mm) is made from the top edge of the top vertebra to the bottom edge of the bottom end vertebra on the concave side of the curve. An identical measurement is made from the same vertebrae on the convex side of the curve. The thicknesses of the intervertebral discs were then measured on the concave and convex sides of the curve. The thicknesses of the discs were summed together on the concave and convex sides, and then subtracted from the longitudinal measurement made on the concave and convex sides of the curve. If the subtracted sums were within 5 to 10 mm of one another, then it was assumed that the reconstructed spine would approach “straight” after the discs were removed. If the difference in the subtracted measurements was more than 10 mm, this indicated the need to either add another disc to the preoperative plan or take off bony wedges from the endplates to get the spine straight.

Pre-operative x-rays are analyzed and measured. They can nicely predict the quality of correction which can be obtained during the surgical procedure. This technique was first identified and used early in the series and is extremely reliable (Figures 6, and 7).

### Why use the “stretch film”

The fundamental measurement made on the “stretch film” is the Cobb angle of the major curve while the spine is being stretched. The unique value of the “stretch film” it that is clearly identifies the area of major deformity, while the spinal column is compensated, over the pelvis, and the compensatory curves are well corrected. In the author’s experience, this type film is much easier to interpret than bending films, which, by their nature, are NOT made with the patient’s spine fully compensated and the compensatory curves minimized.

### Analyzing/measuring the “stretch film”

From the Cobb angle on the “stretch film,” a measurement (in mm) is made from the top edge of the top vertebra to the bottom edge of the bottom end vertebra on the concave side of the curve. An identical measurement is made from the same vertebrae on the convex side of the curve. The thicknesses of the intervertebral discs were then measured on the concave and convex sides of the curve. The thicknesses of the discs were summed together on the concave and convex sides, and then subtracted from the longitudinal measurement made on the concave and convex sides of the curve. If the subtracted sums were within 5 to 10 mm of one another, then it was assumed that the reconstructed spine would approach “straight” after the discs were removed. If the difference in the subtracted measurements was more than 10 mm, this indicated the need to either add another disc to the preoperative plan or take off bony wedges from the endplates to get the spine straight.

### Total discectomy (Figures 8, 9, 10 and 11)

**Figure 8:**
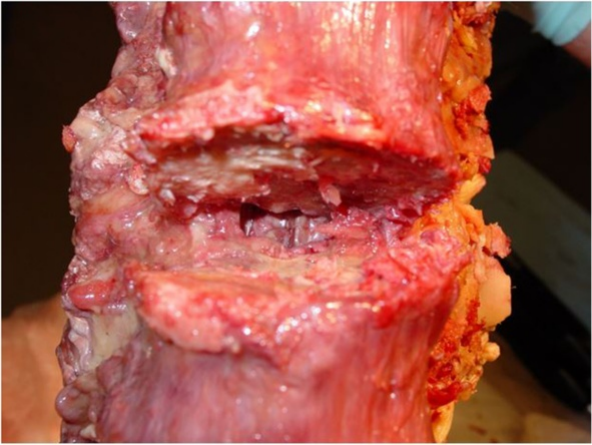
**The entire annulus, including the posterior annulus just anterior to the spinal canal must be removed during “total discectomy”.** This permits “bone-on-bone” apposition which is the unique feature which facilitates the unique short-segment correction of the flexible curves which are ideal for this approach.

**Figure 9:**
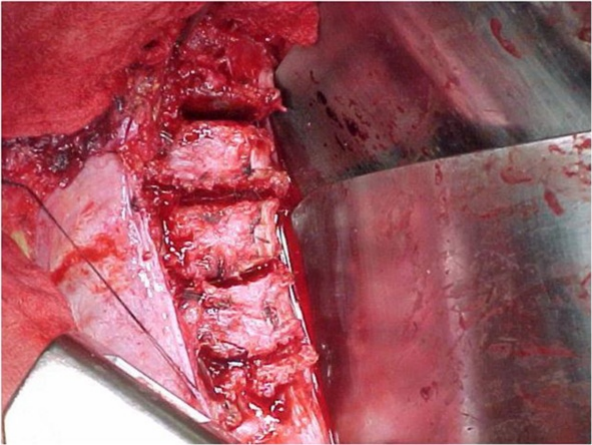
**This 56 degree curve is almost straight and no implants exist yet.** All of the correction has occurred as a consequence of “total discectomies” at each interspace. The overwhelming majority of correction of the curve occurs this way, during properly performed “bone-on-bone” procedures. The implants just achieve the final 5-10% of the correction, not most of it.

**Figure 10:**
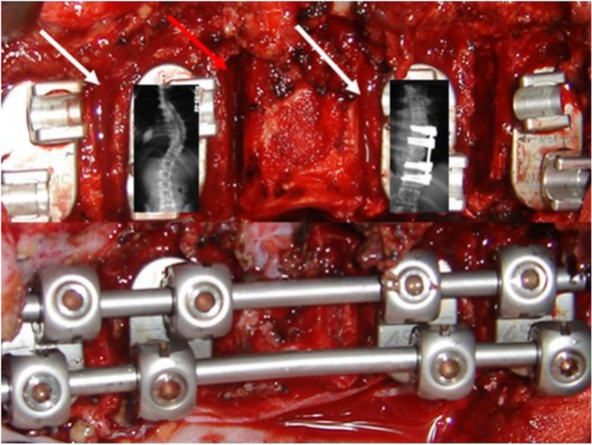
**Illustration demonstrating how little implant application and compression provides to total correction of this 65 degree curve.** Above illustration: discectomies completed staples and screws in place, but no rods. Apposition is almost final, but slight compression must occur at two of the four discs within the construct. Two of the four discs are lying in “bone-on-bone” apposition, following the “total discectomy” at each interspace. Two need slight additional discectomy, before applying the rods and achieving final “bone-on-bone” apposition at all 4 interspaces.

**Figure 11:**
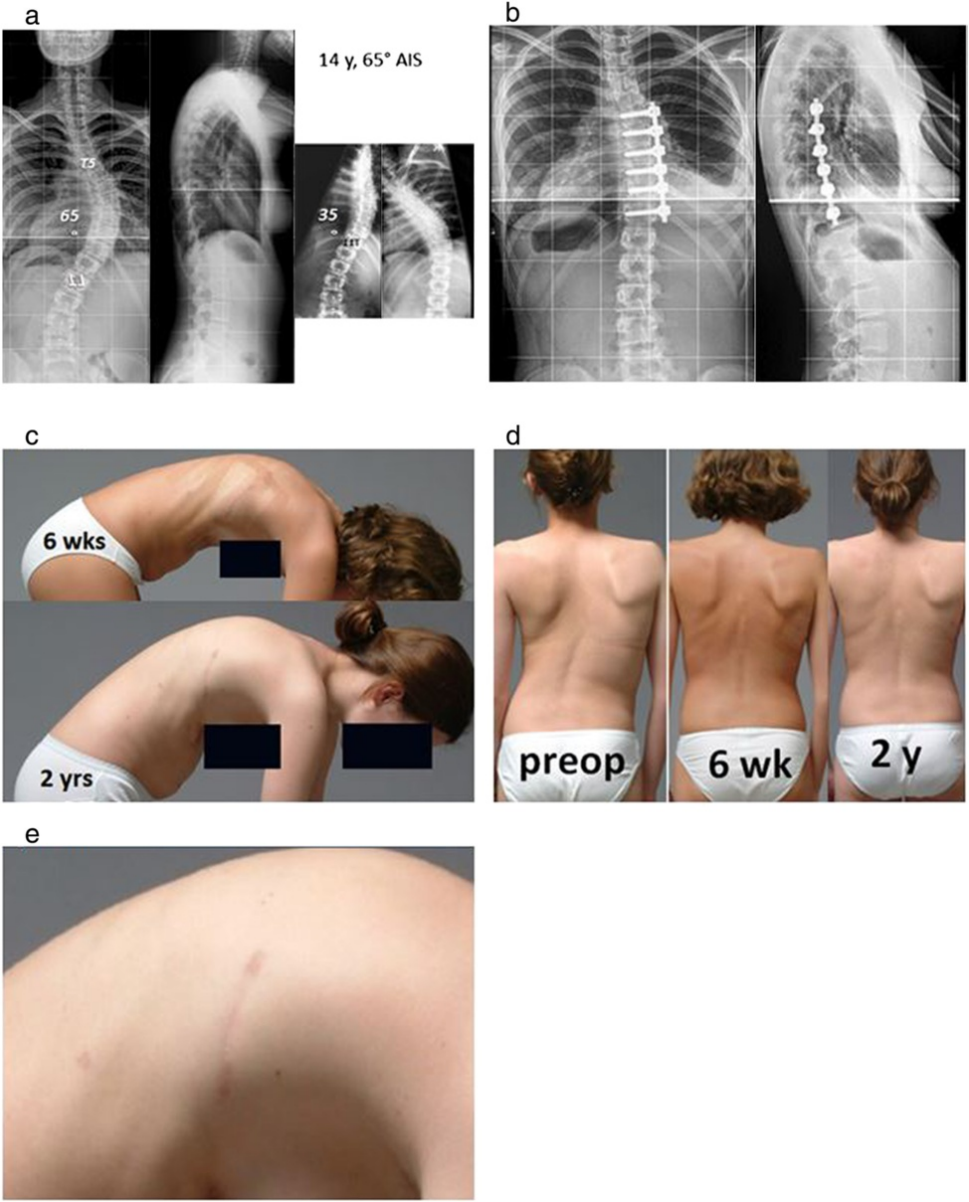
**a: 14 year old girl with 65 degree Lenke 1 curve which bends down to 35 degrees; ideal for “bone-on-bone”. b**: excellent correction in both planes, over 3 fewer fused levels than the measured Cobb angle on the standing film and entire instrumentation within the thoracic spine. It is functionally beneficial for the patient to avoid instrumentation of the thoracolumbar junction if possible, particularly if they have athletic aspirations or will engage in blue collar occupations. **c**: clinical photos of same patient at pre-op, 6 wk. and 2 yr. follow-up of patient in 11a and b. **d**: lateral profile at 6 mos and 2 yrs follow-up. **e**: close-up of healed 8 cm. incision used for the procedure.

#### The mini thoracotomy approach [4]

During the development of anterior spinal reconstruction, open thoracotomy was used. Most surgeons removed most of the exposure rib, and many/most used the exposure rib for bone graft.

Following report of post-op pulmonary function tests which showed some permanent post-operative reduction from normal levels, following open thoracotomy, Newton, Sucato and Lonner began using thoracoscopic partial discectomy and instrumentation for adolescent idiopathic scoliosis to avoid the concern about post-op pulmonary function. After 20 years experience, they abandoned this approach, because of non- union and anterior implant problems. Even with extensive and extended experience, they were not able to reduce the levels of instrumentation necessary for the fusions more than 1 or 2 levels necessary for posterior pedicle-screw instrumentation for identical curves.

**Figure 12:**
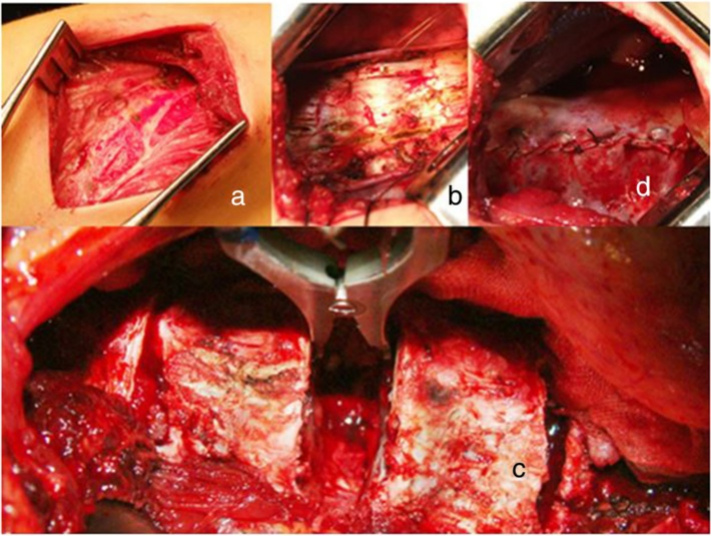
**a: 8 cm incision over the apex illustrated.** 8 cm rib segment removed centered in the mid-auxiliary line. **b**: pleura incised and reflected and retracted with stay sutures. **c**: “total discectomy” performed--identical to open procedure--at all apical levels--identified by the levels included on the “Cobb angle on the stretch film.” **d**: once implants placed, “bone-on-bone” apposition obtained, pleura must be closed over the implants—as illustrated.

**Figure 13:**
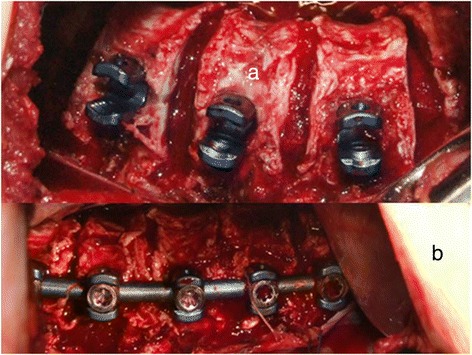
**a: “total discectomy” performed at 4 discs and bicortical screws at 5 vertebrae.** Gelfoam placed into each disc following the discectomy to limit bone oozing. **b**: “bone-on-bone” apposition is obtained by compression, until the apposition is palpable with a Penfield 4 dissector.

Dr. Kan Min developed an open “mini-thoracotomy” approach to perform the “bone-on- bone” reconstruction for appropriately selected patients. He first reported his post- operative pulmonary function and clinical results in 2012 in European Spine Journal. His superb results not only reduced the levels of discectomy and instrumentation to roughly half the levels necessary for posterior instrumentation, but also showed no diminution of post-operative pulmonary functions in his 62 operated patients (Figures 12, 13 and Additional file 1).

### Clinical series

#### Dr Gaines’ experience

The first paper on “bone-on-bone” was published in 2003 by Brodner, Gaines et. al [1]. Our first consecutive 31 patients were presented in this paper. Every patient was followed, the mean follow-up of the patients was 41 months, and each patient had been at least 2 years since their surgery. The patients were happy, and the surgeons were also happy.

No serious complications had occurred, healing was occurring predictably, and the patients had returned to the active lives we anticipated when we began the project.

There were no non-unions, implant fractures or loosening, and no infections and no other serious systemic complications.

In 2009 [2], we published an extended follow-up of these 31 patients and added 14 more, operated in the interval, a total of 45 patients with a mean follow-up of 6 years for the entire group, and minimum follow-up of 28 months for every patient. No patient was lost to follow-up.

Four patients (8.9%) had an incomplete expansion of their operated lung during the early postoperative period. None of them required any treatment for this, and all resolved uneventfully. One patient (2.2%) had a pleural effusion that resolved spontaneously, one (2.2%) had pneumonia, and one (2.2%) had urinary tract infection, treated with antibiotics. Eight patients developed a narcotic ileus (17.8%). All of these postsurgical complications resolved uneventfully.

In this series, as in the first, there were no non-unions, or implant-related complications (breakage or loosening) and no revision surgeries.

By the time this publication appeared, we had gained the extraordinary confidence in the procedure we have today.

Since that report, we have had one patient who was engaged in extraordinarily physical exertion in the Intensive Care Unit on the second post-operative day. She did pull out one level of her anterior implants and had to have a posterior reinforcement of her implants. One posterior level was added to her instrumentation and fusion. This is the only return to the operating room, or re- operation in our entire series of 160 patients in 18 years. There have still been no infections or other serious complications which have delayed recovery.

### Dr. Zarzycki and his group’s experience [3]

Dr. Zarzycki’s experience, accumulated over a decade by his 10 young well trained surgeons, reassured us that the techniques necessary were not unique to surgeons in Missouri. The fact that they were able to reproduce exactly the same excellent results we produced was first published in the European Spine Journal Volume 16, Suppl 1, str. 23 and 96. He analyzed his first 250 consecutive cases for AIS. He was very happy with his results, like we have been.

**Figure 14:**
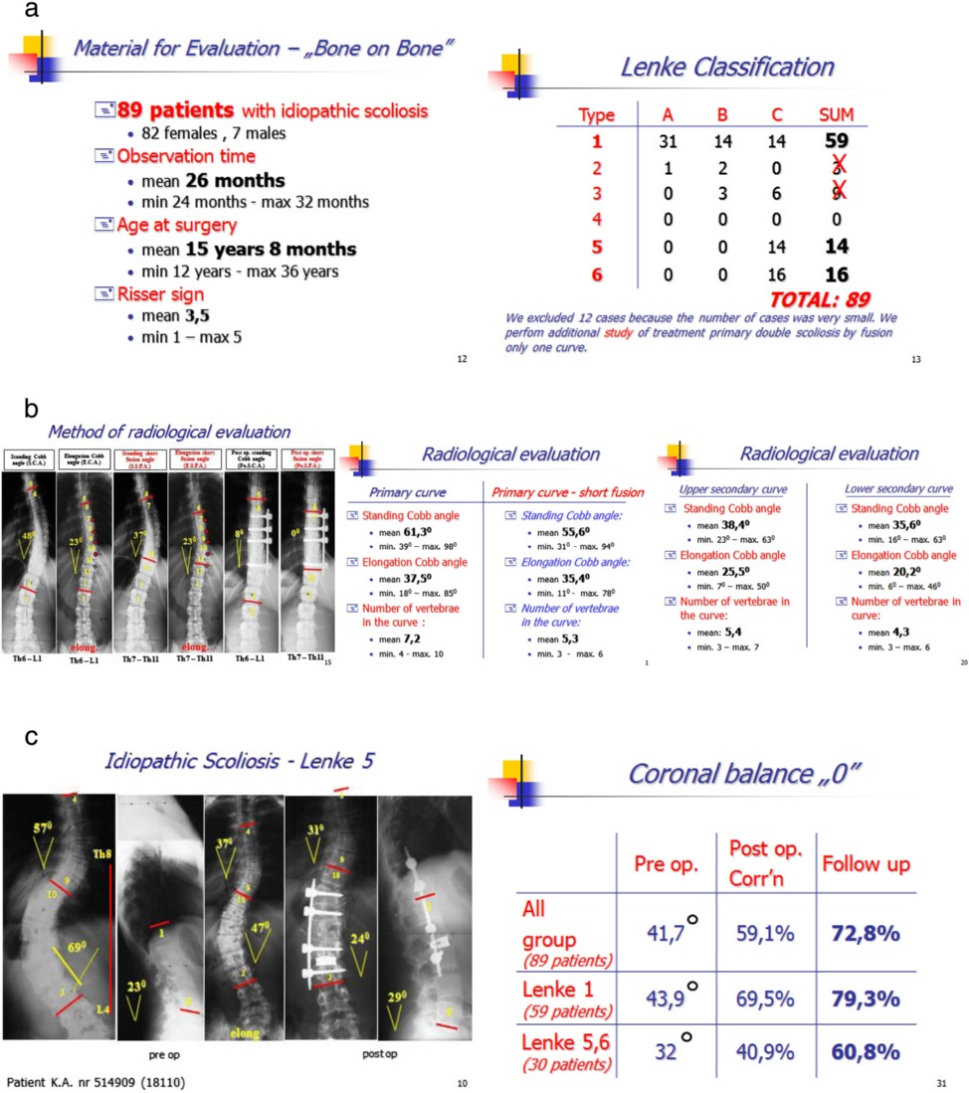
**a: Early data from Dr. Zarzycki’s group presented in 2005, early experience. b**: A typical Lenke 1 case and data regarding Dr. Zarzycki’s group’s early series of Lenke 1 cases, from 2005. **c**: Typical Lenke 5 case and early data presented in 2005 by Dr. Zarzycki and his group.

The fact that this clinical experience has now been extended to over 1300 cases, (personal communication) over the past 10 years, reinforces our confidence in it. The fact that most of his cases were not performed by him, but by his young faculty, further reinforces the fact that “easily learned evaluative and surgical skills” underpin the “bone-on-bone” procedure---NOT ones which can only be performed by “surgical wunderkind.” (Figure 14).

### Dr. Min and the “Mini-Thoracotomy Approach” [4]

When Dr. Min published his “Mini-Thoracotomy Approach” for the procedure, we immediately read his publication and went to Zurich to see it.

His excellent surgical skills and excellent report about his 62 consecutive patients’ “normal” post-operative pulmonary function tests made it obvious that the “bone-on-bone” procedure should adopt the mini-thoracotomy approach.

The mini-thoracotomy approach provides perfectly sufficient exposure to clearly visualize the discs in the fusion area, so “total discectomy” can be performed on each of them, and also sufficient exposure so the implants can easily be placed, “bone-on-bone” apposition created by compression and implant final locking achieved. The pleural can then be closed over the implants.

### Complications

In the past many years, the “bone-on-bone” reconstruction has provided a reliable and very predictable surgical solution for patients with easy, flexible, single curve adolescent idiopathic scoliosis whose curves have gotten into the range where surgical reconstruction is the best treatment option. While the reconstructions have not been “complication-free,” they have been almost “serious complication-free.”

Until the recent case required posterior revision, we had not performed a surgical revision for over 15 years. This unique patient was a true “outlier” and we do not anticipate a recurrence of the events which required her revision again.

We believe the mini-thoracotomy approach has provided the in introduction to a “new era” in the surgical management of AIS which will provide experienced surgeons an efficient way to minimize the fusion levels involved with these fortunate patients so their spines will perform quite well, even in athletic endeavors and/or blue collar occupations.

### Consent

“Informed consent was obtained from each patient included in the study for performance of the procedure and use of images obtained during the patient's treatment.”

## Additional file

Additional file 1:
**Movie illustrating the entire procedure.**
